# Repurposing the McoTI-II Rigid Molecular Scaffold in to Inhibitor of ‘Papain Superfamily’ Cysteine Proteases

**DOI:** 10.3390/ph14010007

**Published:** 2020-12-23

**Authors:** Manasi Mishra, Vigyasa Singh, Meenakshi B. Tellis, Rakesh S. Joshi, Shailja Singh

**Affiliations:** 1 Department of Life Sciences, School of Natural Sciences, Shiv Nadar University, Gautam Buddha Nagar 201314, India; vigyasa105@gmail.com; 2 Special Centre for Molecular Medicine, Jawahar Lal Nehru University, New Delhi 110067, India; 3 Division of Biochemical Sciences, CSIR-National Chemical Laboratory, Dr. Homi Bhabha Road, Pune 411008, India; meenakshikapase@gmail.com (M.B.T.); rs.joshi@ncl.res.in (R.S.J.); 4 Academy of Scientific and Innovative Research (AcSIR), Ghaziabad 201002, India

**Keywords:** cystatin, McoTI-II, cyclotide, protein engineering, Clan CA cysteine proteases, papain, cathepsin

## Abstract

Clan C1A or ‘papain superfamily’ cysteine proteases are key players in many important physiological processes and diseases in most living systems. Novel approaches towards the development of their inhibitors can open new avenues in translational medicine. Here, we report a novel design of a re-engineered chimera inhibitor Mco-cysteine protease inhibitor (CPI) to inhibit the activity of C1A cysteine proteases. This was accomplished by grafting the cystatin first hairpin loop conserved motif (QVVAG) onto loop 1 of the ultrastable cyclic peptide scaffold McoTI-II. The recombinantly expressed Mco-CPI protein was able to bind with micromolar affinity to papain and showed remarkable thermostability owing to the formation of multi-disulphide bonds. Using an in silico approach based on homology modelling, protein–protein docking, the calculation of the free-energy of binding, the mechanism of inhibition of Mco-CPI against representative C1A cysteine proteases (papain and cathepsin L) was validated. Furthermore, molecular dynamics simulation of the Mco-CPI–papain complex validated the interaction as stable. To conclude, in this McoTI-II analogue, the specificity had been successfully redirected towards C1A cysteine proteases while retaining the moderate affinity. The outcomes of this study pave the way for further modifications of the Mco-CPI design for realizing its full potential in therapeutics. This study also demonstrates the relevance of ultrastable peptide-based scaffolds for the development of novel inhibitors via grafting.

## 1. Introduction

Cysteine proteases are one of the four main groups of peptide bond hydrolases found in all forms of life and play regulatory roles in a range of physiological and pathological processes [1,2,3]. They all share a common catalytic mechanism involving a nucleophilic cysteine thiol for peptide bond hydrolysis [4,5]. Clan CA, family C1, is one of the largest and the best characterized subfamilies of cysteine proteases and also known as ‘papain-like cysteine proteases (PLCPs)’ because all members are structurally related to papain. PLCPs have a two-domain structure and their activity depends on a highly conserved catalytic triad (Cys25–His159–Asn175) which forms the substrate-binding pocket/active site cleft located between the domains [3,4,5,6].

Mammalian counterparts of PLCPs are lysosomal cysteine cathepsins (cathepsin B, H, L, C, X, F, O, V) that are involved in normal cellular protein degradation and turnover in mammals [7,8]. Cathepsins have been implicated in the development and progression of many diseases involving abnormal protein turnover and thus, are popular therapeutic targets for several diseases like arthritis, osteoporosis, atherosclerosis, cancer, inflammatory, and immune-related diseases [1,8,9,10,11,12]. PLCPs in parasites are also known for their indispensable roles in parasite growth and cell differentiation, signalling and host invasion [11,13,14,15,16]. Their suitability as a potential drug target for antiparasitic chemotherapy is very well validated [15,17,18]. Therefore, the design and synthesis of cysteine protease inhibitors (CPIs) holds significant value in medicine and biotechnology. Inhibitors offering metabolic stability, high specificity, and membrane penetrability hold greater implication in the context of being more effective in diseases and translation into drugs. Engineered biomimetic peptides which can modulate protein–protein interactions are popular leads among better strategies for therapeutics and targeted drug delivery. Conformationally restricted peptide scaffolds have attracted widespread interest in presenting structured protein domains or specific epitopes in therapeutic applications [19,20,21,22,23,24,25].

Among the widely accepted frameworks for protein engineering has been the plant-derived cysteine-rich cyclotides [26,27]. Cyclotides are head-to-tail cyclic mini proteins (~35 aa) which possess a highly stable cyclic cystine knot (CCK) motif. Owing to the high stability of the CCK motif, they are resistant to proteolytic, thermal, and chemical degradation. Their exceptional stability and tolerance to substitutions in their backbone loops have made them popular molecular scaffolds for protein engineering [23,28]. These peptides are also able to be internalized by mammalian cells by endocytosis owing to their cell-penetration properties [29,30]. Their oral bioavailability, safety, and efficacy profiles in humans have also been validated [31]. Cyclotides like Kalata B1, McoTI-I/II have been widely used to graft peptide sequences for the purpose of presenting novel epitopes with novel biological functions [23,28,32,33,34,35,36]. Therefore, they are of huge interest in targeting intracellular pharmaceutical targets.

McoTI-II is a stable cyclic peptide found in seeds of the plant *Momordica cochinchinensis*. It belongs to the trypsin inhibitor subfamily of cyclotides and has been the most preferred framework for grafting and engineering novel biological activities. Naturally, it exhibits a potent (sub-nM) trypsin inhibitory activity [37,38]. A range of substitutions in the trypsin inhibitory reactive site (loop 1) and the P1 residue have been used to develop a series of McoTI-I analogs with specificity towards alternate proteases with moderate affinity [28]. The development of inhibitors of foot-and-mouth-disease virus 3C protease, matriptase, β-tryptase, Bcr-Abl kinase and FXIIa using loop 1 grafts (typically, 6–8 residues bioactive epitopes) have validated the amenability of loop 1 for sequence variations [23,28,39,40].

In the present work, we tried to seek whether the McoTI-II structure can be re-engineered to exhibit novel inhibitory specificity. We harnessed the natural cystatin–cysteine protease interaction by designing a chimera inhibitor using the McoTI-II ultrastable scaffold and cystatin first hairpin loop conserved Gln-X_aa_-Val-X_aa_-Gly (QxVxG) motif. Cystatins are reversible, tight-binding inhibitors of PLCPs with conserved sequence motifs [41,42,43]. The partially flexible amino terminus, the first β-hairpin loop containing the highly conserved QVVAG region, and the second β-hairpin loop (Pro-Trp) forms a hydrophobic wedge-shaped ‘edge’ which is highly complementary to the active site cleft of papain [41,44]. In the chimera inhibitor, the amino acid substitutions made in the trypsin inhibitory loop 1 of McoTI-II have redirected its specificity towards C1A cysteine proteases.

Here, we report the recombinant expression, biochemical characterization, and activity of the chimera inhibitor (Mco-CPI) for model C1A cysteine protease, papain. Results from the in silico studies based on protein–peptide docking and molecular dynamics simulation demonstrate an effective modulation of McoTI-II scaffold from trypsin inhibitor to a CPI. We also discuss the implications of this study for future work aimed at realizing the full potential of the Mco-CPI inhibitor design strategy for targeting intracellular cysteine cathepsins or parasitic PLCPs for therapeutic applications.

## 2. Results

### 2.1. Modulation of McoTI-II Scaffold for the Design of C1A Cysteine Protease Inhibitor

**Figure 1:**
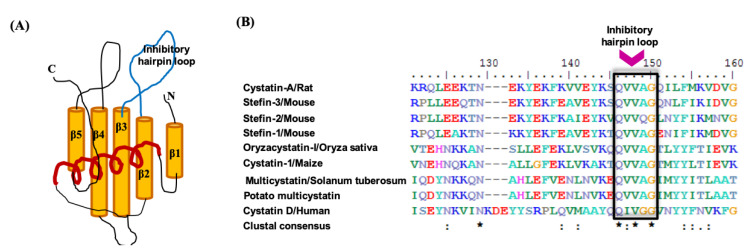
Representation of the cystatin superfamily and its conserved features. (**A**) Diagrammatic representation of the tertiary structure of cystatin protein. This comprises a core of five-stranded anti-parallel β-sheets wrapped around a central α-helix with a conserved inhibitory hairpin loop (QxVxG motif) between β2 and β3. (**B**) Multiple sequence alignment showing the conserved motif in cystatins from human cystatin D (PDB: 1RN7_A); Stefin-1 (Uniprot:P35175), stefin-2 (Uniprot: P35174), stefin-3 (Uniprot: P35173) from Mouse, cystatin-A (Uniprot:P01039) from rat; Oryzacystatin-I (PDB: 1EQK_A) from *Oryza sativa*; Cystatin I (Uniprot:P31726) from *Zea mays*; Multicystatin (Uniprot: P37842), (PDB: 2W9P_F) from *Solanum tuberosum*. Clustal consensus is shown below and the asterisk (*) represents the conserved residue. The conserved inhibitory hairpin loop motif (QxVxG) is shown in a box.

The tertiary structure of cystatin protein inhibitors comprises a core of five-stranded anti-parallel β-sheets wrapped around a central α-helix as shown in Figure 1A. Multiple sequence alignment of the amino acid sequences of cystatins: Cystatin D from human, Stefins from mouse, Cystatin A from rat; Oryzacystatin-I from *Oryza sativa*; Cystatin I from *Zea mays*; and Multicystatin from *Solanum tuberosum* show high conservation and adherence to the conserved protein domain ‘cystatin’ family (pfam00031) (Supplementary Figure S1 and Figure 1B). The Gln-Val-Gly (Q-X-V-X-G) motif in the inhibitory hairpin loop (IHL), known for playing the main interactions with cysteine proteases, is conserved in all sequences (Figure 1B). Therefore, we used this conserved motif sequence (QVVAG) as a ‘graft’ to incorporate in the ultrastable, cysteine-rich scaffold of McoTI-II.

**Figure 2:**
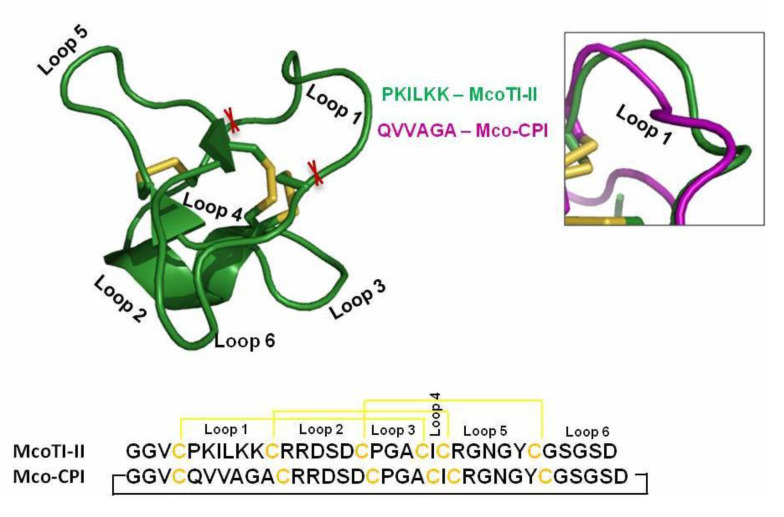
Design of C1A cysteine protease inhibitor based on the MCoTI-II scaffold. Amino acid sequence and 3D structure of McoTI-II (PDB: 1IB9) displaying its typical disulphide bonding and the cyclic structure. The loop 1 sequence (PKILKK) was substituted by cystatin loop sequence (QVVAGA) as a ‘graft’ in design of Mco-cysteine protease inhibitors (CPIs) while the rest of the sequence remains identical. The inset shows the superimposition of loop 1 of both McoTI-II (green) and Mco-CPI (purple).

McoTI-II is a knottin family potent trypsin inhibitor harbouring the inhibitory activity site (PKILKK) within loop 1, bounded by two cysteine residues. Therefore, we incorporated our graft sequence (QVVAGA) in loop 1 for a potential design of a C1A cysteine protease inhibitor. The tertiary structure of the chimera inhibitor Mco-CPI generated from the Raptor X server based on its closest homologs in PDB exhibited overall similarity with the McoTI-II structure (Figure 2 and Supplementary Figure S2). The Mco-CPI structure showed the formation of three disulphide bonds C1–C4, C2–C5, C3–C6 as typical of cyclotide scaffold (Figure 2) providing them their remarkable rigidity. The substituted loop 1 in Mco-CPI also appeared mostly alike to loop 1 of McoTI-II in its orientation as observed in the superimposition of the NMR structure of McoTI-II (PDB: 1IB9) and Mco-CPI (Figure 2). However, the orientation of the side chain residues in loop1 is different because of the substituting amino acid residues. The superposition of the predicted structure of Mco-CPI on the template structure of McoTI-II using Cα atom positions gave a root-mean-square deviation (RMSD) of 1.58 and a TM-score of 0.57440 which is indicative of both the structures having the same fold [45]. Therefore, the loop 1 substitutions in McoTI-II do not alter its tertiary structure from its typical cyclotide fold.

### 2.2. Recombinant Production and Biochemical Characterization of Mco-CPI

**Figure 3:**
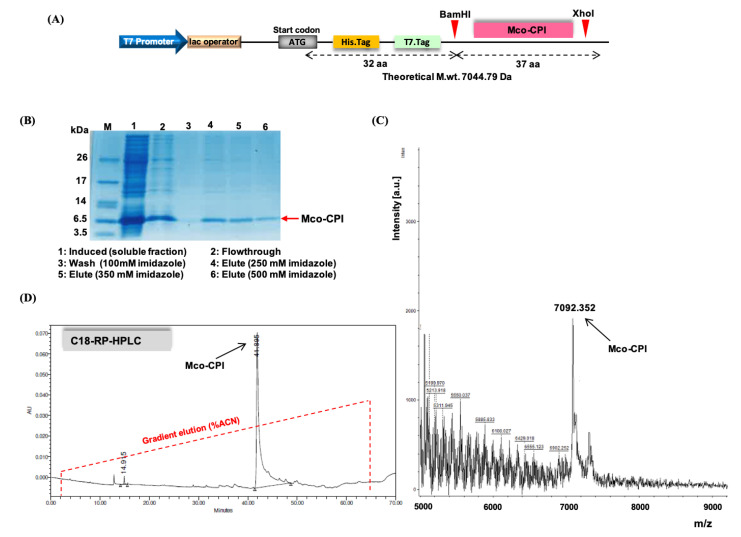
Expression and biochemical characterization of Mco-CPI. (**A**) Diagrammatic representation of the gene construct in pET28a (+) vector for the recombinant expression of Mco-CPI in *E. coli*. Mco-CPI has been cloned between BamHI and XhoI restriction enzyme sites and is preceded by 6XHis Tag and T7 tag sequences. The theoretical m.wt. of protein (with tags) is ~7044.79 Da. (**B**) Ni-NTA purification of Mco-CPI. Purified protein (~7 kDa) separated on 15% SDS-PAGE (tris-tricine) and stained with Coomassie blue R250. (**C**) MALDI-TOF–MS spectrum of Mco-CPI showing a peak of 7092.352 Da. (**D**) Analytical RP-HPLC trace of Mco-CPI showing a single major peak with ~ 41 min retention time (with ~50% acetonitrile) is indicative of its hydrophobic property.

The schematic representation of the design of the construct for recombinant expression is shown in Figure 3A. The 114 bp coding sequence of Mco-CPI (37 aa) cloned between BamHI and XhoI restriction enzyme sites is preceded by 6XHis Tag and T7 tag (Supplementary Figure S3). Therefore, the encoded Mco-CPI protein has plus 32 aa incorporating the two tags and theoretical m.wt. upcoming to 7044.79 Da. For the recombinant protein expression, *E. coli* Shuffle 30 cells were chosen as they are engineered for the production of correctly disulphide-bonded active proteins in high yields in the cytoplasm [46]. This is a *trxB gor* suppressor strain where cytoplasmic reductive pathways are diminished and also constitutively express a chromosomal copy of disulphide bond isomerase, Disulfide bond C (DsbC), which assists in the formation of correctly folded multi-disulphide-bonded proteins [46]. Protein expression conditions were optimized at varying temperatures and induction conditions. Mco-CPI protein showed an enhanced solubility at lower growth rates (at 25 °C for 12–16 h post-induction) (Figure 3B). The Mco-CPI protein in the soluble fraction was purified on the basis of His Tag (Ni-NTA affinity) and the final protein was equivalent to ~7 kDa on SDS-PAGE (Tris-tricine) (Figure 3B).

The purified recombinant Mco-CPI protein was further analysed by a combination of reverse phase-high performance liquid chromatography (RP-HPLC) and matrix-assisted laser desorption–ionization/time of flight-mass spectrometry (MALDI-TOF–MS) to determine its hydrophobic properties and accurate molecular mass, respectively. The mass spectrum was acquired in the range of 1–10 kDa (6–10 kDa range for enhanced visibility). A prominent peak of ~ 7092 Da was observed which is in accordance with the SDS-PAGE protein profile and the expected theoretical mass of Mco-CPI (Figure 3C). The aliphatic index and grand average of hydropathicity (GRAVY index) of Mco-CPI as calculated by its amino acid composition are 38.12 and −0.507, respectively, indicating that the protein exhibits hydrophobicity to some extent. The hydropathicity score for most of the proteins range from −2 to +2, the more positively scored proteins being more hydrophobic [47]. The analytical RP-HPLC analysis of Mco-CPI resulted in the late elution of a single major peak at ~41 min retention time with ~50% acetonitrile (Figure 3D). A prediction based on the amino acid composition by the Kyte and Doolittle hydrophobicity scale is also indicative of the hydrophobic nature of the protein which is mostly because of the cyclotide scaffold (Supplementary Figure S4).

The predicted structure of Mco-CPI suggested that there is a formation of three disulphide bonds (Supplementary Figure S2). We performed the Ellman’s assay for the experimental estimation of free cysteines which would thereby infer on the disulphide bonding within Mco-CPI [48]. Based on the standard thiol SH (L-cysteine; 5–100 µM) calibration curve, the free thiol concentration for L-cysteine is 18.34 (mol/mol) and for Mco-CPI it was calculated as 0.222 (mol/mol) (Supplementary Figure S5), indicating the almost full absence of free thiols in Mco-CPI. This result supports the prediction that all six cysteines are most likely to be involved in disulphide bond formation and recombinantly produced Mco-CPI should be in accordance with the predicted structure. The formation of disulphide bonds is essential for the folding, function, and stability of Mco-CPI. However, the limitation of this method is that the actual pairing of cysteines is not validated.

A similar design of construct with McoTI-II sequence was also used for recombinant protein expression and characterization to have a starting control for this protein preparation and comparison of modulated inhibitory specificity (Supplementary Figure S6A,B). The encoded McoTI-II protein with tags had theoretical m.wt. of 7227.14 Da. The McoTI-II protein purified from the soluble fraction showed a band equivalent to ~7.2 kDa on SDS-PAGE and a prominent peak of 7278.79 Da on MALDI-TOF–MS analysis (Supplementary Figure S6C,D).

### 2.3. Inhibitory Activity and Kinetic Analysis of Mco-CPI

**Figure 4:**
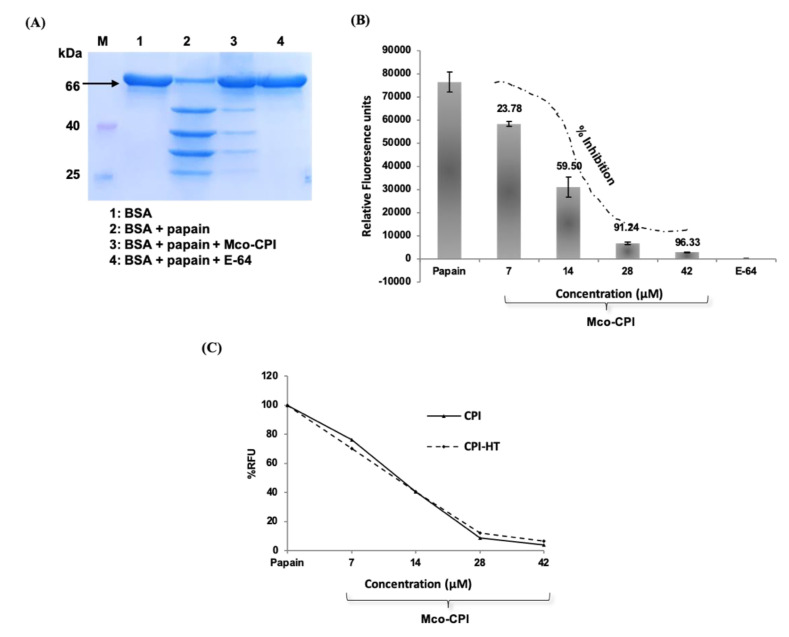
Inhibitory activity of Mco-CPI against papain and inhibitor stability. (**A**) Proteolytic degradation of BSA (at 66 kDa, marked by black arrow) by papain in the presence and absence of Mco-CPI as checked on SDS-PAGE. E-64 was used a positive control to monitor the complete inhibition of papain digestive activity on BSA. Mco-CPI could inhibit the papain proteolytic activity partially within the given time. (**B**) Inhibition kinetics of Mco-CPI against papain. The inhibition of papain follows a sigmoidal pattern with the increasing concentration of the inhibitor, indicating tight binding. (**C**) Papain inhibition with increasing concentrations of Mco-CPI and heat-treated Mco-CPI. The data were plotted as % relative fluorescence units (RFU) assuming papain fluorescence as 100% and compared with the not treated sample. There is no decrease in the inhibitory activity upon heat-treatment and it is indicative of the thermostability of Mco-CPI.

A preliminary proteolytic activity assay was done to validate the inhibitory ability of the Mco-CPI protein against cysteine proteases (papain, here) using a natural protein substrate, bovine serum albumin (BSA) (Figure 4A). The reaction mix included 0.1% sodium dodecyl sulphate (SDS) to reduce the secondary structure of the substrate and did not inactivate the enzyme, papain. SDS-treated BSA (~66 kDa) was almost completely digested to products of Mr ~55, 40, 30, and 25 kDa by papain within 8 min (Figure 4A). The pre-incubation of papain with E-64 (synthetic inhibitor) could completely inhibit the proteolytic activity of papain with no digestion products observed on the gel. E-64 is an epoxide which is known as a potent irreversible inhibitor of cysteine proteases [49]. It interacts with the *S*n subsites of the cysteine proteases via a covalent bond formation. E-64 and its derivatives exhibit IC_50_ values in the nanomolar range against papain and thus is widely used as a broad-spectrum inhibitor of PLCPs [49]. On the pre-incubation of papain with Mco-CPI protein, most of the BSA remained undigested with very faint digestion fragments observed on the gel. This result suggested the inhibitory activity of Mco-CPI against papain.

Furthermore, the kinetic analysis revealed the strong inhibition of papain by Mco-CPI recombinant protein (IC_50_, 11.72 µM). The inhibition of papain followed a sigmoidal pattern with an increasing concentration of the inhibitor protein, suggesting a reversible and competitive inhibition with tight binding (Figure 4B). A similar mode of inhibition against trypsin was observed for the McoTI-II protein with an IC_50_ of 2.12 µM (Supplementary Figure S6E). This result indicated that the amino acid substitutions made in loop 1 have altered the specificity of McoTI-II to papain. The inhibition constant Ki (~6.776 µM) and the experimental free energy of binding (∆G_exp_ = −6.716 kcal mol^−1^) was determined based on the dose-dependent response data, which suggest the tight and thermodynamically spontaneous binding of Mco-CPI with papain. The Mco-CPI protein retained its inhibitory activity even after heat treatment (80 °C for 10 min). Similar concentrations of the heat-treated inhibitor did not show any decrease in their inhibitory activity against papain (Figure 4C). These data back up the stability of the inhibitor due to the formation of disulphide bonds as predicted in in silico studies and Ellman’s assay.

### 2.4. Mco-CPI Interaction with Model C1A Cysteine Proteases: Papain and Cathepsin L

**Figure 5:**
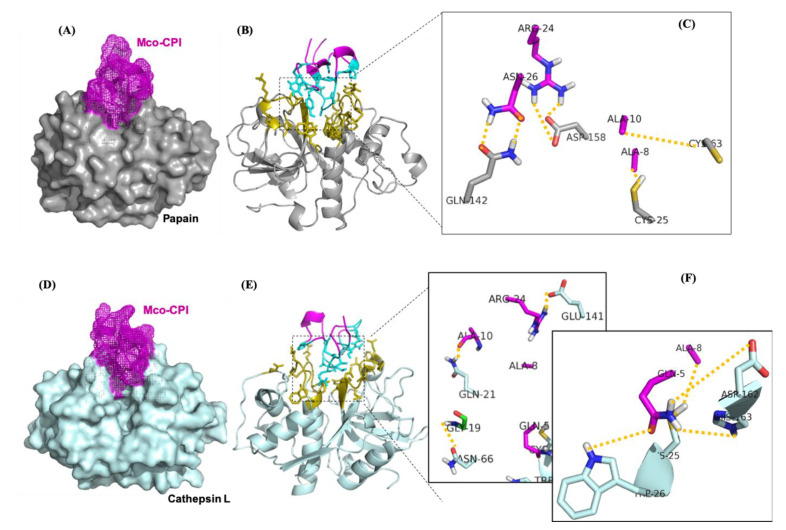
C1A cysteine proteases in a complex with Mco-CPI. (**A**) Surface representation of the Mco-CPI–papain complex (purple-gray). (**B**) The interface residues of papain (olive) and Mco-CPI (blue). (**C**) The important residues at the interface involved in hydrogen bonding (represented by dotted yellow lines). (**D**) Surface representation of the Mco-CPI–cathepsin L complex. (**E**) The interface residues of cathepsin L (olive) and Mco-CPI (blue). (**F**) The residues at the interface involved in hydrogen bonding. Residues in the catalytic diad/triad (CYS-25, HIS-163) form important contacts with the ALA-8 and GLN-5 of the inhibitory loop 1 in Mco-CPI. All figures have been prepared in PyMOL.

The molecular mechanism of interaction between Mco-CPI and papain was predicted using protein–protein docking studies. Protein–protein docking generates as many near-native complex structures as possible. The HADDOCK server clustered 168 structures in 11 clusters, representing 84% of the water-refined models. The scores, energies, RMSD, and buried surface area of all clusters are summarized in Supplementary Table S1. The selected Mco-CPI–papain complex model (based on the highest score) showed that Mco-CPI fits into the active site cleft of papain through its inhibitory loop (i.e., loop 1) with an interface area (Å^2^) of 559 for papain (6%) and 646 for the inhibitor (25%) (Figure 5A,B, Table 1). The interface residues are illustrated in Figure 5B. The molecular model predicted several atomic interactions of the inhibitory loop 1 with the catalytic triad and also between interface the residues of both interacting proteins. In the Mco-CPI–papain interaction, the carbonyl oxygen atom of ALA-8 and amino nitrogen of ALA-10 of Mco-CPI loop1 formed hydrogen bonds with side chains of CYS-25 and CYS-63 in papain, respectively. Sidechains of ARG-24 and ASN-26 of Mco-CPI formed hydrogen bonds with GLN-142 and ASP-158 of papain, respectively (Table 2, Figure 5C). Additionally, ASP-158 in papain also formed two salt bridges with two hydrogens of ARG-24 in Mco-CPI. These results illustrated interactions of the catalytic diad residues and the subsite residues known for substrate binding in papain to be in interaction with Mco-CPI loop 1 residues and other interface residues. The prediction of the free energy of binding between Mco-CPI and papain indicated the formation of a stable complex with Δ*G*_bind_ to be −10.7 kcal mol^−1^. These values are comparable in magnitude to the experimental free energy of binding (∆G_exp_ = −6.716 kcal mol^−1^). These results show substrate-like binding with papain and its inhibition by Mco-CPI, similar to what was observed in inhibition assays.

Cysteine cathepsins also belong to the clan CA and C1 family of papain-like enzymes. Thus, as a representative, cathepsin L interactions with Mco-CPI were also predicted using the molecular docking approach. HADDOCK server clustered 151 structures in 11 clusters, representing 75% of the water-refined models. The scores, energies, RMSD, and buried surface area of all clusters is summarized in Supplementary Table S2. The Mco-CPI–cathepsin L complex model showed substrate-like fitting of Mco-CPI into the active site cleft of cathepsin L with an interface area (Å^2^) of 642 for cathepsin L (6.5%) and 716 for the inhibitor (28%) (Figure 5D,E, Table 1). The interface residues are illustrated in Figure 5E. In Mco-CPI–cathepsin L interaction, the carbonyl oxygen atom of ALA-8 and ALA-10 of Mco-CPI loop1 formed hydrogen bonds with side chains of CYS-25 and GLN-21 in cathepsin L, respectively. GLN-5 of Mco-CPI forms hydrogen bonds with TRP-26, ASP-162, HIS-163 of cathepsin L. GLY-19 and ARG-24 of Mco-CPI form hydrogen bonds with ASN-66 and GLU-141 in cathepsin L, respectively (Table 3, Figure 5F). Salt bridge interaction is also observed between ARG-24 and GLU-141. The prediction of the free energy of binding between Mco-CPI and cathepsin L indicated strong binding with Δ*G*_bind_ of −10.4 kcal mol^−1^. These results illustrate the similar binding mechanism of Mco-CPI with two representatives of the clan CA cysteine proteases.

### 2.5. Molecular Dynamics Simulation of Mco-CPI–Papain Complex

**Figure 6:**
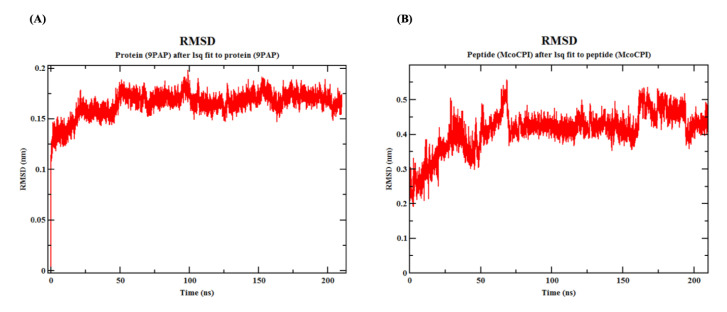
Molecular dynamics simulation of the Mco-CPI–papain complex. (**A**) Root-mean-square-deviation (RMSD) of papain (PDB ID: 9PAP) over the simulation of 210 ns. (**B**) RMSD of peptide (Mco-CPI) over the simulation of 210 ns. This indicates that the individual proteins within this interaction remain stable.

**Figure 7:**
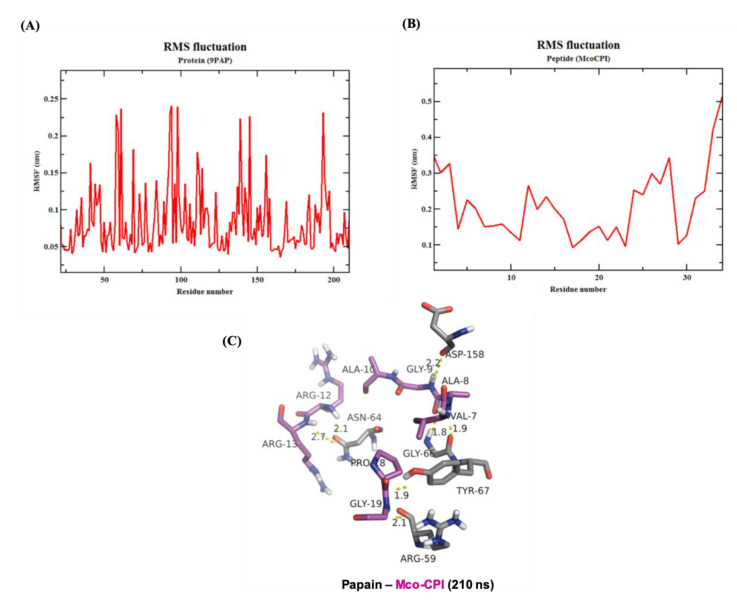
Molecular dynamics simulation of the Mco-CPI–papain complex. (**A**,**B**) RMSF of papain (PDB ID: 9PAP) and Mco-CPI residues over the simulation of 210 ns. (**C**) Intermolecular hydrogen bonds in the Mco-CPI–papain complex at the frame of 210 ns.

Furthermore, the molecular dynamics (MD) simulation of the Mco-CPI–papain complex over 210 ns indicated that the interaction remains stable (Figure 6 and Figure 7). The MD simulation yielded a total energy of −5.42892 kJ/mol and a potential energy of −6.7336 kJ/mol for the Mco-CPI–papain complex. The root-mean-square-deviation (RMSD) of papain shows the initial fluctuation, but later remains stabilized (0.15 to 0.2 nm) throughout the simulation (Figure 6A). The RMSD of the inhibitor peptide (Mco-CPI) shows more fluctuation initially up to 70 ns, then attains stability and deviates between 0.3 and 0.5 nm (Figure 6B).

The root-mean-square-fluctuation (RMSF) analysis of the Mco-CPI–papain complex indicated that the interacting residues in the complex remains stable over the simulation (Figure 7). The RMSF of papain residues ranges from 0.05 to 0.25 nm over the simulation range while the RMSF of Mco-CPI residues ranges from 0.1 to 0.3 nm (Figure 7A,B). The RMSF of loop1 residues (within Mco-CPI) lies between 0.15 and 0.2 nm, signifying that the reactive loop remains stable within the complex structure. The stability of the overall complex is also evident by the similar number of hydrogen bonds observed in the frame of 210 ns (Figure 7C).

## 3. Discussion

In order to design a novel peptide inhibitor of clan CA cysteine proteases, we used McoTI-II as the molecular scaffold (Figure 2). In McoTI-II (Uniprot entry P82409), Lys10 (within loop 1) occupies the P1 position in the substrate-binding site and is the key determinant of its trypsin inhibitory activity (Figure 2). The substitution of this residue and other loop 1 residues is, therefore, expected to alter the protease specificity thereby, enabling the development of inhibitors of alternative proteases [28]. Therefore, we grafted the cystatin conserved inhibitory hairpin loop sequence (QVVAG) within loop 1 (Figure 1A,B). Out of the three conserved motifs in cystatin (see Introduction), this loop is proven to behave in a substrate-like manner and interact readily with the PLCPs [44]. Several studies using this loop sequence to develop synthetic substrates for PLCPs and derivatized peptide inhibitors of PLCPs have suggested the suitability of this motif as a pseudo-substrate/inhibitor [50,51,52]. Furthermore, this sequence substitution in loop 1 would not alter the typical CCK motif of the McoTI-II scaffold, which was validated by the predicted structure of the chimera inhibitor Mco-CPI (Figure 2).

Mco-CPI was recombinantly produced using the pET vector system in *E. coli* Shuffle 30 cells, which are specialized for the formation of correctly folded multi-disulphide bonded proteins. This expression system including the N-terminal tags enabled the production of protein in the soluble fraction and its facilitated purification (Figure 3). The characterization of the purified Mco-CPI protein by RP-HPLC and MALDI-TOF-MS confirmed the ≥90% purity of the recombinant protein (Figure 3), and multi-disulphide bonding was inferred by free thiol content estimation (Supplementary Figure S5). However, in our protein preparation, the presence of tag residues increased the relative size of the Mco-CPI protein and the ideal cyclic topology of Mco-CPI remains compromised due to the absence of the backbone cyclization. The backbone cyclization plays a very important role in the stability of the molecule as an inhibitor and thus its affinity to the target. The chemoenzymatic ligation of backbone or intein-mediated protein trans-splicing methods [53,54,55,56] are used for the production of cyclic scaffolds, both being beyond the capacity of our resource outreach at present. Hence, to validate our design (substitutions in loop 1) in principle against the C1A cysteine proteases, we performed kinetic assays with our recombinant Mco-CPI preparation.

Kinetic assays showed a competitive mode of inhibition and strong binding with the papain of the recombinant Mco-CPI (IC_50_, 11.72 µM; Ki ~6.776 µM) (Figure 4B). The McoTI-II recombinant protein preparation used in our study as a starting control (Supplementary Figure S6) showed trypsin inhibition in the micromolar range (IC_50_, 2.12 µM) as compared to the sub-nM range reported for McoTI-II purified from the plant sources or chemically synthesized as a cyclic molecule [37,38]. Therefore, we expect that a modification involving a backbone cyclization would lead to further improvement in the efficiency and stability of Mco-CPI against papain. A similar mechanism of inhibition of papain was validated by the molecular docking studies which indicated the fitting of loop 1 into the active site cleft of papain in a substrate-like manner owing to its complementarity and strong binding (Δ*G*_bind_ −10.7 kcal mol^−1^) (Figure 5). The catalytic diad residues (CYS-25, HIS-159) and the subsite residue for substrate binding (ASP-158) show strong interactions with the loop 1 residues (ALA-8) and interface residues (ARG-24) of the Mco-CPI (Figure 5). In order to confirm this mechanism of inhibition of Mco-CPI against PLCPs, we chose another popular representative cathepsin L for molecular docking studies. In the Mco-CPI–cathepsin L model, catalytic diad residues (CYS-25, HIS-163) and subsite residues (TRP-26, ASP-162) also showed strong interactions with the loop 1 residues (GLN-5, ALA-8, ALA-10) and interface residues (ARG-24) of the Mco-CPI (Figure 5). These interactions are typical of the cystatin/stefin-papain complex. MD simulation of the Mco-CPI–papain complex also validated that the intermolecular interactions within this complex remain stable (Figure 6 and Figure 7).

To conclude, we can affirm that in this study, the grafting of cystatin IHL residues in loop 1 had successfully altered the specificity of McoTI-II from trypsin to cysteine protease (C1A) inhibition. However, we do apprehend that the inhibitory effect and stability like cyclic topology might not have been achieved by Mco-CPI protein in in vitro assays. However, this study validates this novel design and paves the way for the development of cyclic Mco-CPI using chemical synthesis methods for its improved efficacy in disease models. We also do speculate that cyclic Mco-CPI, owing to its ultrastability, cell-penetrability, and non-cytotoxicity, would be of high relevance in therapeutics.

## 4. Materials and Methods

### 4.1. Production of Gene Construct

A synthetic gene encoding for chimera inhibitor MCo-CPI was synthesized and cloned in pET-28a(+) vector by GenScript (Genscript Biotech, Piscataway, NJ, USA) (Supplementary Figure S3A,B). The Mco-CPI coding sequence was optimized for expression in *Escherichia coli*. The coding sequence (114bp) of MCo-CPI has been cloned between BamHI and XhoI restriction enzyme sites and is preceded by 6XHis Tag and T7 tag sequences. The complete nucleotide sequence of the open reading frame (start codon to stop codon) and its translation is illustrated in Supplementary Figure S3C. The similar design of the construct for the recombinant expression of McoTI-II is shown in Supplementary Figure S6A,B.

### 4.2. Recombinant Expression and Purification of Inhibitor

The construct plasmid was used to transform the Shuffle 30 strain of *E. coli* and transformants were selected on Luria-Bertani medium (LB) plates with kanamycin as selection marker. A single colony containing Mco-CPI-pET28a (+) was grown in LB medium up to mid-log phase (OD = 0.5 at 600 nm) and then induced with 0.3 mM isopropyl β-D-1-thiogalactopyranoside (IPTG). Cells were further grown for 16 h at 25 °C. Cells were harvested at 12,000 rpm at 4 °C. Pellets were washed with ice-cold 50 mM Na_2_HPO_4_, 150 mM NaCl (pH 7.2), 10 mM imidazole, sonicated (five cycles of 5 s each, with cooling for 30 s between the cycles), and centrifuged at 12,000 rpm for 30 min at 4 °C. The soluble protein fraction was used for further purification.

For the purification of the recombinant protein, the supernatant was incubated 2–3 h at 4 °C with Ni-NTA resin (Qiagen) pre-equilibrated with 50 mM Na_2_HPO_4_, 150 mM NaCl (pH 7.2), 10 mM imidazole. The column was washed with ten-bed volumes of the same buffer having 50 mM imidazole, and the bound protein was eluted with a continuous gradient (250–500 mM) of imidazole. Purified protein was analysed with 15% SDS-PAGE (Tris-tricine) and BCA protein quantitation assay (Pierce BCA protein assay kit, Thermofisher Scientific, Waltham, MA, USA). Purified protein aliquots were dialyzed against 20 mM phosphate buffer and desalted using Amicon Ultra centrifugal filters (MWCO, 3 kDa). The final protein preparation (~7 kDa) was with 6xHIS Tag and T7 tag. Protein was stored in aliquots at −20 °C for further characterization.

### 4.3. Biochemical Characterization of Mco-CPI Protein

#### 4.3.1. HPLC

Mco-CPI was diluted in 1 mL of HPLC buffer A (H_2_O, 0.1% trifluoroacetic acid (TFA)) and analysed by HPLC using an isocratic of 100% buffer A for 2 min and then a linear gradient of 5% to 95% buffer B (90% acetonitrile in H_2_O, 0.1% TFA) in 65 min. Flow rate was set a 0.3 mL/min and detection at 220 and 280 nm. All solvents used were HPLC grade (Merck Millipore, Sigma, St. Louis, MO, USA). C18 reverse phase HPLC column (Ascentis C18 HPLC column, 5 µm particle size, I. × I.D. 15 cm × 4.6 mm, SUPELCO, Sigma) was used on an analytical HPLC system equipped with gradient capability and UV–vis detection (Waters Corporation).

#### 4.3.2. MALDI-TOF–MS

MALDI-TOF analysis was done using MALDI TOF/TOF Mass Spectrometer (Ultraflextreme, Bruker Daltonics at KSBS, IIT, New Delhi, India). Samples were spotted 1:1 with freshly prepared matrix (sinapinic acid in 30% acetonitrile, 0.1% TFA) directly onto a stainless steel MALDI target plate. The MALDI-TOF spectra were acquired in a positive reflector mode with the following parameters: source voltage 20 kV, mass range 1000–10,000 Da, focus mass ~7000 Da, and 50 single laser shots over each sample spot.

#### 4.3.3. Free Thiol Content by Ellman’s Assay

The standard curve for L-cysteine (Sigma Aldrich) was done for the concentration range of 5 µM to 150 µM using standard Ellman’s assay protocol [57]. Fifty microliters (50 µL) of the sample was mixed with 950 µL of 5,5′-dithiobis-(2-nitrobenzoic) acid (DTNB) working reagent (prepared by mixing 50 µL of DTNB solution, 100 µL of Tris-HCl (pH 8.0) and, 800 µL of MQ-water). The mixture was incubated at 37 °C for 10 min and absorbance was measured at 412 nm. The concentration of free thiol in the unknown sample (i.e., MCo-CPI) was calculated as

Free thiol concentration (M) = Absorbance/(path length × e) × dilution factor

where e is the extinction coefficient of DTNB = 14, 150 M^−1^ cm^−1^. The free thiol concentration was then divided by the protein concentration to express the values as free thiol per Mco-CPI (mol/mol) [58].

### 4.4. Inhibitory Activity of Mco-CPI

#### 4.4.1. Proteolytic Activity Assay

The protein substrate, BSA, at a concentration of 1.0 mg/mL in 0.1 M sodium acetate buffer (pH 5.5) containing 0.1% SDS was incubated with papain, papain preincubated with E-64, and papain preincubated with Mco-CPI protein. The papain enzyme used was pre-activated with 2 mM dithiothreitol. The ratio of enzyme to the substrate (E:S) was maintained at 1:10. The digestion was allowed to be carried at 40 °C for 8 min. This method was adapted from Zucker et al., 1985 [59]. The degradation products of each reaction were analysed on 12% SDS-PAGE gel.

#### 4.4.2. Inhibitory Activity Assays

The activity and inhibition of papain by the Mco-CPI protein were estimated using 7-amino-4-methyl coumarin (AMC) substrate in a spectrofluorometric assay. Papain (Sigma Aldrich) (21.5 µM) was incubated with and without Mco-CPI at different concentrations (7, 14, 28, and 42 µM) in 50 mM sodium acetate at pH 5.5, 8 mM DTT for 30 min, followed by the addition of substrate (50 uM, Z-Phe-Arg-7-amino-4-methyl coumarin). The release of the AMC group was monitored for 10 min at room temperature at an excitation of 360 nm and emission 465 nm. E-64 was used as a positive control to monitor the complete inhibition of papain. The data were plotted as relative fluorescence units (RFU) (at endpoint) vs. concentration (µM). Three technical replicates were performed for all assays. The IC_50_ was calculated using the Quest Graph^TM^ IC_50_ calculator which models the dose-dependent response dataset using a logistic regression model and resolves as a sigmoid function. Ki value was calculated directly from IC_50_ values using the Cheng–Prusoffs classical equation, K_i_ = IC_50_/(1 + [S]/K_m_) [60]. The experimental binding energy (∆G_exp_) was approximated using the equation, ∆G_exp_ = −RTlnIC_50_ where R is the gas constant, and T is the temperature in kelvin [61]. The inhibition of trypsin by McoTI-II was also estimated similarly using the ZFR-AMC substrate in buffer conditions: 50 mM Tris-HCl pH 8.0, 50 mM NaCl, 5 mM CaCl_2_. PMSF (1 mM) was used as a positive control to monitor the complete inhibition of trypsin (Sigma Aldrich).

#### 4.4.3. Thermostability and Residual Inhibitory Activity

Mco-CPI protein was heat-treated at 80 °C for 10 min. The treated samples were then used for inhibition assays with papain as described above. The data were plotted as % RFU assuming papain fluorescence as 100% and compared with the not treated samples.

### 4.5. Molecular Modeling and Docking with Target C1A Cysteine Proteases: Papain, Cathepsin L

Molecular interactions between Mco-CPI and target proteases, papain and cathepsin L were predicted using protein–protein docking studies. The structure of Mco-CPI was predicted by homology modelling based on its highest ~90% sequence identity with an engineered cyclotide called MCo-PMI (PDB ID: 2M86) which is based on McoTI-I scaffold. A 3D model was generated using the structure prediction tool Raptor X [62]. The quality of the model was assessed using PROCHECK, RAMPAGE, and ProSA analysis [63,64].

This model was used for protein–protein docking with papain (PDB ID: 9PAP) and human cathepsin L (PDB ID: 6F06), selected as representatives of C1A cysteine proteases. A docking study was conducted to evaluate the binding conformation, interaction, and binding energy of Mco-CPI with papain and cathepsin L. For both Mco-CPI–papain and Mco-CPI–cathepsin L, respectively, 3D models were submitted to the HADDOCK server for docking with standard input parameters [65]. The primary interactive residues were specified for Mco-CPI (Gln5, Val6, Val7, Ala8, Gly9, and Ala10); the papain catalytic triad (Cys25, His159, Asn175) and cathepsin L catalytic diad (Cys25, His163). The best generated docking model was analysed for the binding pose, interface residues, intermolecular covalent and non-covalent interactions using the COCOMAPS tool and PDBsum analysis with a cut off for a distance of atom–atom interaction = 5Å. The binding affinity and dissociation constant were predicted using PRODIGY [66,67].

### 4.6. MD Simulations

The Mco-CPI–papain docked complex was used for MD simulations. All the MD simulations were performed using the Charmm36-jul2017 force field with GROMACS 5.1.4 package [68,69], (GROMACS User Manual version 5.1.4). During the MD simulations, all the protein atoms were surrounded by a cubic water box of 8 × 8 × 8 (nm) of TIP3 water molecules. The net charge on the system was zero, so no ions were added explicitly. Energy minimization was performed using the steepest descent algorithm. The system was equilibrated under NVT conditions for 100 ps followed by an NPT equilibration of 1000 ps. The velocity-rescaling algorithm was applied to maintain the system temperature (300 K) and Parrinello–Rahman pressure coupling was used to maintain the pressure at 1 bar. A production run of 210 ns with a time step of 2 fs was performed. RMSD calculations were done for papain (9PAP) and peptide (Mco-CPI) over the complete simulation range.

## Figures and Tables

**Table 1:** Predicted intermolecular interactions and interface statistics (cut off for the distance of atom–atom interaction = 5 Å) of Mco-CPI–papain and Mco-CPI–cathepsin L complexes.

	No. of Interface Residues	Interface Area (Å^2^), (%)	No. of Hydrogen Bonds	No. of Salt Bridges	Free Energy of Binding (ΔG) in Kcal mol^−1^
Papain	25	558.9, 6	6	2	−10.7
Mco-CPI	15	646.5, 25.3			
Cathepsin L	25	642.2, 6.5	7	2	−10.4
Mco-CPI	17	716.2, 28.1			

**Table 2:** Predicted intermolecular hydrogen bonds in the Mco-CPI–papain complex.

Papain	Dist (Å) H-Bonds	Mco-CPI
CYS, 25 (HG)	1.70	ALA, 8 (O)
GLN, 142 (HE22)	1.87	ASN, 26 (OD1)
CYS, 63 (O)	3.10	ALA, 10 (N)
ASP, 158 (OD2)	1.89	ARG, 24 (HH12)
ASP, 158 (OD2)	1.78	ARG, 24 (HH22)
GLN, 142 (OE1)	2.02	ASN, 26 (HD22)

**Table 3:** Predicted intermolecular hydrogen bonds in the Mco-CPI–cathepsin L complex.

Cathepsin L	Dist (Å) H-Bonds	Mco-CPI
CYS, 25 (HG)	1.74	ALA, 8 (O)
TRP, 26 (N)	3.49	GLN, 5 (OE1)
HIS, 163 (ND1)	3.04	GLN, 5 (O)
GLN, 21 (HE21)	2.00	ALA, 10 (O)
ASP, 162 (O)	2.49	GLN, 5 (HE22)
ASN, 66 (OD1)	3.12	GLY, 19 (N)
GLU, 141 (OE1)	1.54	ARG 24, (HH11)

## Supplementary Materials

The following are available online at https://www.mdpi.com/1424-8247/14/1/7/s1. Supplementary Figure S1: Multiple sequence alignment of cystatins; Supplementary Figure S2: Predicted tertiary structure of inhibitor Mco-CPI; Supplementary Figure S3: Construct design for recombinant expression of Mco-CPI protein; Supplementary Figure S4: Kyte and Doolittle hydrophobicity plot of Mco-CPI; Supplementary Figure S5: Calibration curve for standard SH for free thiol content estimation by Ellman’s assay; Supplementary Figure S6: Design of construct, recombinant expression and activity of McoTI-II; Supplementary Table S1: Statistics of the top 10 clusters generated for docking of Mco-CPI with papain; Supplementary Table S2: Statistics of the top 10 clusters generated for the docking of Mco-CPI with cathepsin L.

## Abbreviations

PLCPs	Papain-like cysteine proteases
IHL	Inhibitory hairpin loop
CCK	Cyclic cystine knot
McoTI-II	*Momordica cochinchinensis* trypsin inhibitor-II
ZFR-AMC	Z-Phe-Arg-7-amino-4-methyl coumarin
MALDI-TOF-MS	Matrix-assisted laser desorption–ionization/time of flight–mass spectrometry
RP-HPLC	Reverse phase-high performance liquid chromatography
SDS-PAGE	Sodium dodecyl sulphate–polyacrylamide gel electrophoresis
