# Supplementary material for: Repurposing the McoTI-II Rigid Molecular Scaffold in to Inhibitor of ‘Papain Superfamily’ Cysteine Proteases
Source: Pharmaceuticals (Basel). 2020 Dec 23;14(1):7. doi: 10.3390/ph14010007 (PMC7822474; doi:10.3390/ph14010007)
Supplement: Supplementary file 1 [file pharmaceuticals-14-00007-s001.pdf]

Supplementary Figure S1: Full multiple sequence alignment of the amino acid sequences of cystatins from animals and plants [human cystatin D (PDB:1RN7_A); Stefin-1 (Uniprot:P35175), stefin-2 (Uniprot: P35174), stefin-3 (Uniprot: P35173) from Mouse, cystatin-A (Uniprot:P01039) from Rat; Oryzacystatin-I (PDB: 1EQK_A) from *Oryza sativa*; Cystatin I (Uniprot:P31726) from *Zea mays*; Multicystatin (Uniprot: P37842), (PDB: 2W9P_F) from *Solanum tuberosum*].

```
                     10        20        30        40        50        60
             ....|....|....|....|....|....|....|....|....|....|....|....|
sp|P0103     ------------------------------------------------------------
sp|P3517     ------------------------------------------------------------
sp|P3517     ------------------------------------------------------------
sp|P3517     ------------------------------------------------------------
pdb|1EQK     ------------------------------------------------------------
sp|P3172     ----------------------------------------MRKHRIVSLVAALLVLLALA
sp|P3784     MAIVGGLVDVPFENKVEFDDLARFAVQDYNQKNDSSLEFKKVLNVKQQIVAGIMYYITFE
pdb|2W9P     ------------------------------------------------------------
1RN7_A|P     ------------------------------------------------------------
Clustal

                     70        80        90       100       110       120
             ....|....|....|....|....|....|....|....|....|....|....|....|
sp|P0103     -----------------------------MDPGTTGIVGGVSEA--KPATPEIQEVADKV
sp|P3517     -----------------------------MSQENLKIKGGLSEA--RPATPEIQMIADKV
sp|P3517     -----------------------------MTEYTRKIKGGLSEA--RPATSEIQEIADKV
sp|P3517     -----------------------------MS------LGGVSEA--SRATPEIQMIANKV
pdb|1EQK     -----------------------------MSSDGGPVLGGVEPV-GNENDLHLVDLARFA
sp|P3172     AVSSTRSTQKES-----------------VADNAGMLAGGIKDVPANENDLQLQELARFA
sp|P3784     ATEGGNKKEYEAKILLRKWEDLKKVVGFKLVGDDSTMPGGIVNV-PNPNNTKFQELARFA
pdb|2W9P     ---------------------------------------GIVNV-PNPNNTKFQELARFA
1RN7_A|P     ---------------------------GSASAQSRTLAGGIHAT--DLNDKSVQRALDFA
Clustal                                             *:  .        .      .

                    130       140       150       160       170       180
             ....|....|....|....|....|....|....|....|....|....|....|....|
sp|P0103     KRQLEEKTN---EKYEKFKVVEYKSQVVAGQILFMKVDVGNGRFLHMKVLRG---LSGDD
sp|P3517     RPLLEEQTN---EKYEKFEAVEYKSQVVAGQNLFIKIDVGNGCFLHMKVFRG---LSGED
sp|P3517     RPLLEEKTN---EKYEKFKAIEYKVQVVQGLNYFIKMNVGRGCYLHINVLSG---ISSEN
sp|P3517     RPQLEAKTN---KKYEKFEAVEYKTQVVAGENIFIKMDVGHGCFIHIKVFNG---PTGKD
pdb|1EQK     VTEHNKKAN---SLLEFEKLVSVKQQVVAGTLYYFTIEVKEGD-AKKLYEAK---VWEKP
sp|P3172     VNEHNQKAN---ALLGFEKLVKAKTQVVAGTMYYLTIEVKDGE-VKKLYEAK---VWEKP
sp|P3784     IQDYNKKQN---AHLEFVENLNVKEQVVAGIMYYITLAATDDAGKKKIYKAK---IWVKE
pdb|2W9P     IQDYNKKQN---AHLEFVENLNVKEQVVAGIMYYITLAATDDAGKKKIYKAK---IWVKE
1RN7_A|P     ISEYNKVINKDEYYSRPLQVMAAYQQIVGGVNYYFNVKFGRTTCTKSQPNLDNCPFNDQP
Clustal          :   *         : :    *:* *   ::.:        :            .

                    190       200       210
             ....|....|....|....|....|....|.
sp|P0103     DLKLLDYQTNKTKNDELTDF-----------
sp|P3517     DLKLKGYQTNKTKTDELTSM-----------
sp|P3517     DLELTGYQTNKAKNDELTYF-----------
sp|P3517     NYELHGYQTDKTMDEELTYF-----------
pdb|1EQK     WMDFKELQEFKPVDASANA------------
sp|P3172     WENFKQLQEFKPVEEGASA------------
sp|P3784     WEDFKKVVEFKLVG-----------------
pdb|2W9P     WEDFKKVVEFKLV------------------
1RN7_A|P     KLKEEEFCSFQINEVPWEDKISILNYKCRKV
Clustal        .       :
```

Supplementary Figure S2: Predicted tertiary structure of the chimera inhibitor Mco-CPI.

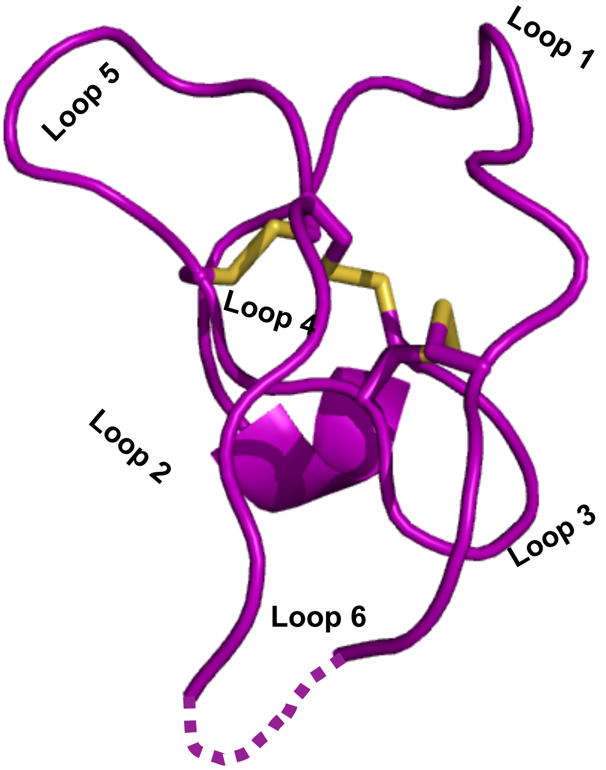

McoCPI **GGVCQVVAGACRRDSDCPGACICRGNGYCGSG**

Supplementary Figure S3: Construct design (in pET-28a(+) vector) for recombinant expression of Mco-CPI protein.

[A]

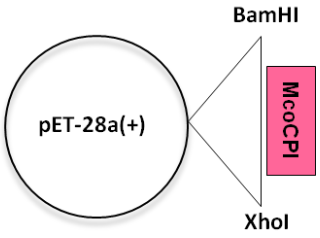

[B]

McoCPI (114 bp)

GGTGGCGTGTGCCAAGTGGTTGCGGGTGCGTGCCGTCGTGACAGCGATTGTCCGGGT
G G V C Q V V A G A C R R D S D C P G

GCGTGCATCTGCCGTGGTAACGGCTACTGCGGTAGCGGCAGCGACAACCACGTTTAA
A C I C R G N G Y C G S G S D N H V *

[C]

Nt sequence

ATGGGCAGCAGCCATCATCATCATCATCACAGCAGCGGCCTGGTGCCGCGCGGCAGCCATATGGCTAGCATGACTGGTGGA
CAGCAAATGGGTCGC**GGTGGCGTGTGCCAAGTGGTTGCGGGTGCGTGCCGTCGTGACAGCGATTGTCCGGGTGCGTGCAT**
CTGCCGTGGTAACGGCTACTGCGGTAGCGGCAGCGACAACCACGTTTAA

Translation: Amino acid sequence

MGSSHHHHHHSSGLVPRGSHMASMTGGQQMGR **GGVCQVVAGACRRDSDCPGACICRGNGYCGSGSDNHV**

Supplementary Figure S4: Kyte & Doolittle hydrophobocity plot of Mco-CPI. The more positive the value, the more hydrophobic are the amino acids located in that region of the protein.

MGSSHHHHHHSSGLVPRGSHMASMTGGQQMGR **GGVCQVVAGACRRDSDCPGACICRGNGYCGSGSDNHV**

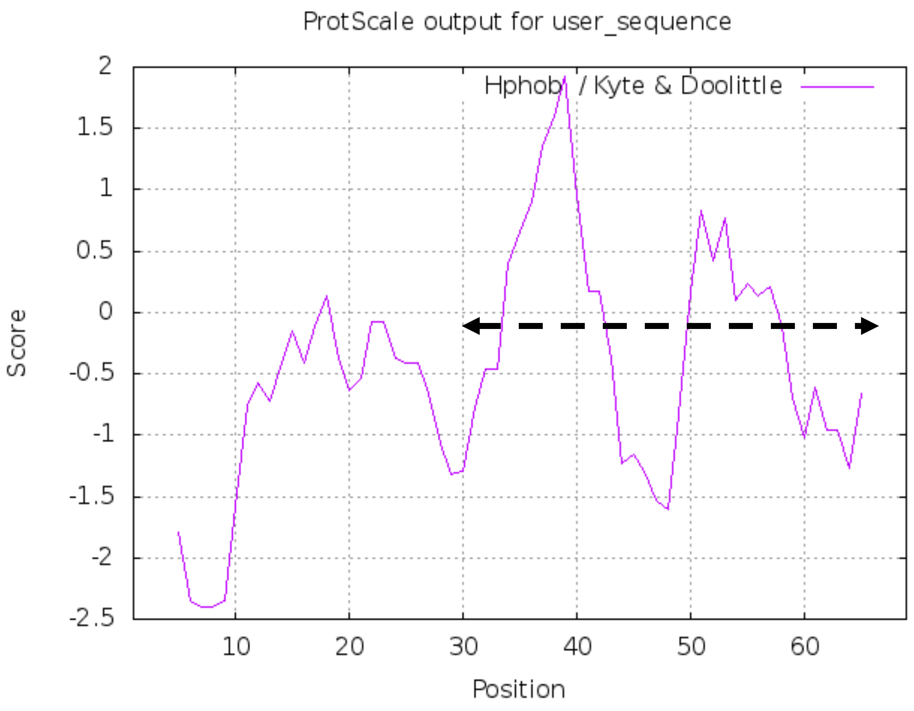

Supplementary Figure S5: Calibration curve for standard SH (L-cysteine; 5-100 μM) for free thiol content estimation by Ellman's assay.

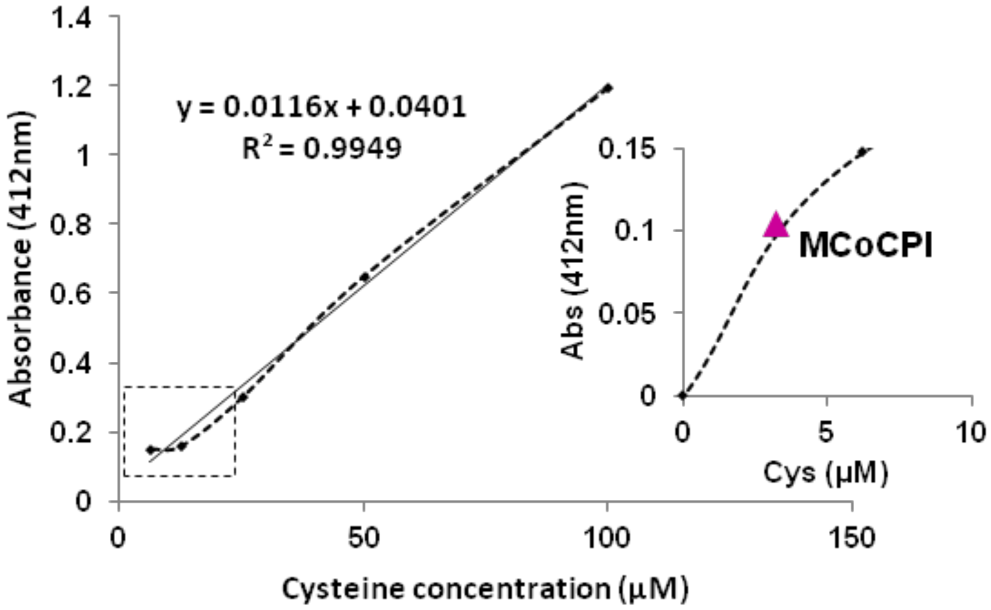

Supplementary Figure S6: Construct design and characterization for McoTI-II protein. **A) & B)** Construct design and nucleotide sequence encoding McoTI-II. **C)** MALDI-TOF-MS spectrum of recombinant McoTI-II showing a peak of 7278.795 Da. Also, at a difference of 150 Da, a peak of 7126.421 is evident. This could be the result of terminal processing of an amino acid during expression. **D)** Ni-NTA purification of McoTI-II. Purified protein (~7.2 kDa) separated on 15% SDS-PAGE (tris-tricine) and stained with coomasie blue R250. **E)** Inhibition kinetics of McoTI-II against trypsin expressed as % trypsin inhibition. An increase in % trypsin inhibition is observed with increasing concentration of McoTI-II.

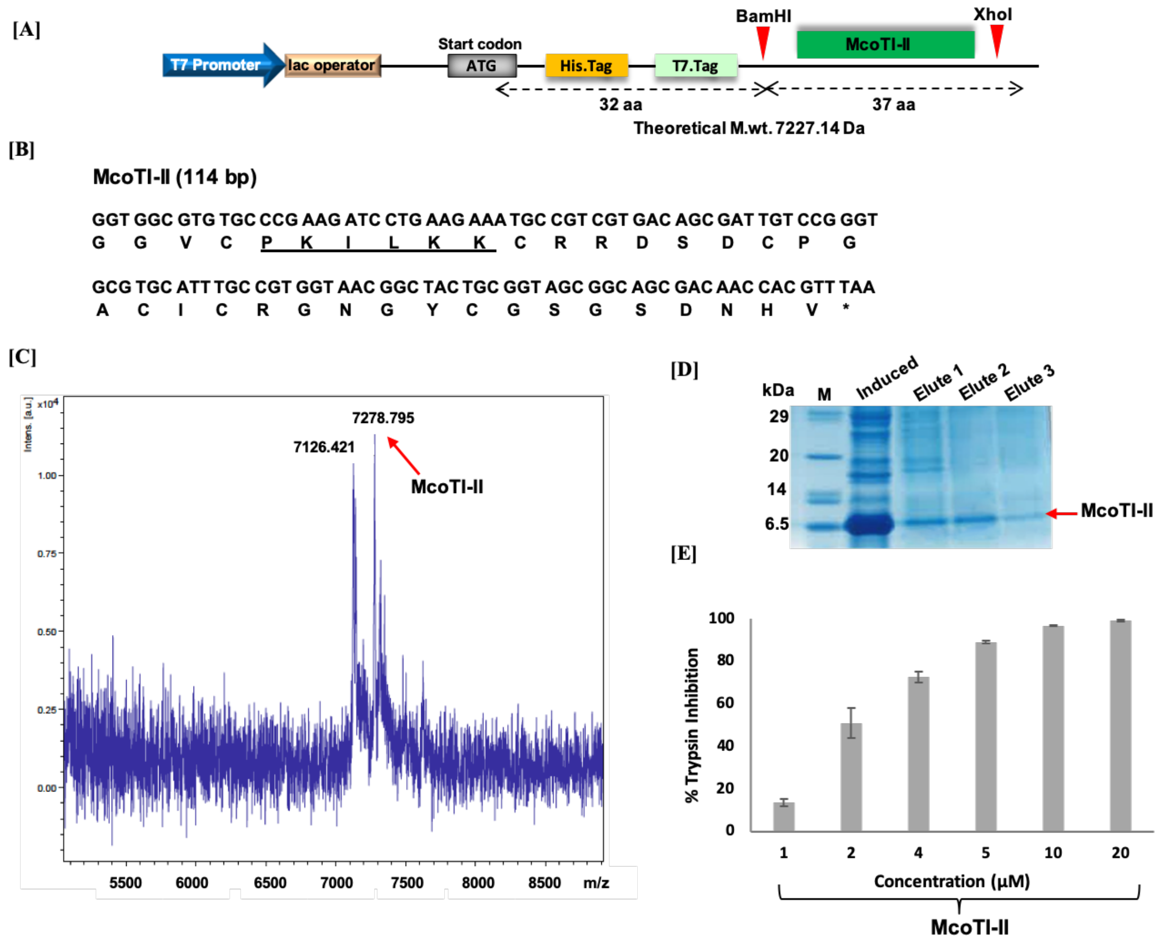

Supplementary Table 1: Statistics of the top 10 clusters generated for docking of McoCPI with Papain on HADDOCK server

Cluster No.	HADDOCK score	Cluster size	RMSD from overall lowest-energy structure	Van der Waals energy	Electrostatic energy	Desolvation energy	Buried surface area	Z-score
Cluster 3	-66.8 +/- 5.1	17	0.5 +/- 0.4	-43.5 +/- 4.3	-64.9 +/- 4.3	-10.9 +/- 2.3	1184.9 +/- 43.4	-1.5
Cluster 2	-65.5 +/- 3.2	36	7.2 +/- 0.2	-41.1 +/- 5.3	-119.7 +/- 9.1	-1.8 +/- 0.6	1207.3 +/- 45.5	-1.4
Cluster 1	-62.5 +/- 2.5	54	5.5 +/- 0.1	-35.9 +/- 1.7	-107.9 +/- 34.4	-6.3 +/- 3.1	1118.0 +/- 30.8	-1.1
Cluster 9	-55.6 +/- 6.4	5	5.6 +/- 0.2	-38.5 +/- 5.3	-72.0 +/- 24.7	-5.2 +/- 2.7	1105.1 +/- 64.4	-0.5
Cluster 11	-48.6 +/- 4.7	4	7.2 +/- 0.1	-34.8 +/- 3.0	-70.0 +/- 27.9	-3.9 +/- 1.5	940.1 +/- 30.3	0.2
Cluster 4	-48.5 +/- 0.7	16	3.9 +/- 0.2	-36.5 +/- 2.2	-41.0 +/- 8.9	-6.0 +/- 1.2	955.8 +/- 24.6	0.2
Cluster 7	-43.1 +/- 3.4	6	5.2 +/- 0.1	-32.1 +/- 2.3	-37.6 +/- 19.3	-9.1 +/- 3.9	1050.3 +/- 53.7	0.7
Cluster 5	-39.7 +/- 4.2	12	5.3 +/- 0.1	-28.4 +/- 3.0	-41.0 +/- 19.0	-6.3 +/- 5.3	932.5 +/- 26.6	1.0
Cluster 10	-38.0 +/- 14.7	4	4.0 +/- 0.8	-33.0 +/- 10.2	-30.7 +/- 7.7	-2.5 +/- 2.7	926.5 +/- 139.8	1.1
Cluster 8	-36.7 +/- 5.5	5	5.3 +/- 0.2	-27.8 +/- 3.3	-46.8 +/- 24.6	-1.8 +/- 2.0	867.8 +/- 66.7	1.3

Supplementary Table 2: Statistics of the top 10 clusters generated for docking of McoCPI with Cathepsin L on HADDOCK server

Cluster No.	HADDOCK score	Cluster size	RMSD from overall lowest-energy structure	Van der Waals energy	Electrostatic energy	Desolvation energy	Buried surface area	Z-score
Cluster 2	-80.6 +/- 0.8	36	4.9 +/- 0.1	-46.7 +/- 2.6	-147.5 +/- 13.6	-4.5 +/- 1.5	1324.9 +/- 51.7	-1.7
Cluster 1	-74.1 +/- 5.7	45	5.2 +/- 0.3	-49.0 +/- 5.8	-136.7 +/- 10.3	1.9 +/- 1.8	1231.4 +/- 29.5	-1.3
Cluster 3	-68.9 +/- 3.9	11	2.8 +/- 0.1	-44.9 +/- 1.7	-121.4 +/- 23.0	-0.2 +/- 1.1	1268.9 +/- 21.9	-0.9
Cluster 5	-63.5 +/- 5.0	10	4.9 +/- 0.0	-33.9 +/- 4.9	-164.7 +/- 10.9	3.0 +/- 1.4	1184.6 +/- 49.9	-0.5
Cluster 7	-54.2 +/- 1.8	8	5.0 +/- 0.1	-31.7 +/- 3.1	-85.5 +/- 15.7	-6.4 +/- 1.7	1036.5 +/- 54.9	0.1
Cluster 9	-52.9 +/- 7.8	6	5.0 +/- 0.5	-27.8 +/- 5.6	-136.9 +/- 42.0	1.3 +/- 2.8	1012.6 +/- 71.0	0.2
Cluster 6	-46.6 +/- 1.9	10	4.9 +/- 0.1	-35.8 +/- 0.5	-61.8 +/- 12.9	1.3 +/- 1.1	955.6 +/- 58.4	0.6
Cluster 4	-40.2 +/- 10.0	10	5.0 +/- 0.1	-17.8 +/- 3.8	-122.8 +/- 38.1	2.0 +/- 2.6	836.9 +/- 103.2	1.1
Cluster 10	-39.0 +/- 3.6	5	4.6 +/- 0.0	-22.2 +/- 2.2	-87.5 +/- 29.6	-0.7 +/- 1.1	1011.5 +/- 57.4	1.2
Cluster 11	-39.0 +/- 6.1	4	5.0 +/- 0.1	-23.0 +/- 2.7	-74.7 +/- 22.8	-1.2 +/- 2.7	924.4 +/- 144.9	1.2
